# Shear Bond Strength of Self-Adhesive and Self-Etching Resin Cements to Dentin for Indirect Restorations

**DOI:** 10.3390/jfb16080289

**Published:** 2025-08-12

**Authors:** Janet Kirilova, Georgi Veselinov Iliev, Sevda Yantcheva, Elitsa Deliverska, Viktoria Petrova

**Affiliations:** 1 Department of Conservative Dentistry, Faculty of Dental Medicine, Medical University, 1000 Sofia, Bulgaria; janetkirilova@gmail.com (J.K.); s.yancheva@fdm.mu-sofia.bg (S.Y.); 2 Department of Prosthetic Dental Medicine, Faculty of Dental Medicine, Medical University, 1000 Sofia, Bulgaria; g.iliev@fdm.mu-sofia.bg; 3 Department of Dental, Oral and Maxillofacial Surgery, Faculty of Dental Medicine, Medical University, 1000 Sofia, Bulgaria; elitsa.deliverska@fdm.mu-sofia.bg

**Keywords:** resin cement, 10-Methacryloyloxydecyl dihydrogen phosphate, indirect aesthetic restorations, shear strength test

## Abstract

This study assessed and compared the shear bond strength of self-adhesive and self-etching resin cements for indirect aesthetic restorations to dentin. Four different materials, lithium disilicate ceramics, zirconia ceramics, polymethyl methacrylate (PMMA) composites, and hybrid materials, were used for indirect restorations cemented to dentin. The null hypothesis was that there would be no differences in shear bond strength between the investigated materials. Eighty extracted human molars were used. Eighty dentin specimens with a flat surface were prepared and randomly distributed in groups of 10 (n = 10). From each material (Cerasmart 270, Initial LiSi Blok, Katana ZR Noritake, and Crowntec Next Dent), 20 blocks were made and cemented to the dentin samples. Half of the blocks from each material were cemented to dentin using self-etching resin cement (Panavia V5), and the other half using self-adhesive resin cement (i-CEM). After the specimens were prepared, a laboratory test was conducted to evaluate the shear bond strength. The fracture type was determined using a light microscope, and SEM confirmed the results. The results were statistically analysed. All materials cemented with self-etching cements (Panavia V5) showed statistically higher shear strength values than those cemented with self-adhesive resin cement (i-CEM). In the specimen groups where self-adhesive cement (i-CEM) was used, Cerasmart 270 bonded statistically better. A statistical difference was found between all groups of materials cemented with self-etching cement. The Initial LiSi Block showed the strongest bond, followed by Katana Zr Noritake, Crowntec NextDent, and Cerasmart 270. Adhesion fracture to dentin was observed for all groups cemented with i-CEM. This study highlights the superior performance of self-etching cements in terms of shear bond strength. 10-Methacryloyloxydecyl dihydrogen phosphate (10-MDP), a functional monomer, was found to enhance adhesion strength significantly. However, using self-adhesive cements was associated with a weaker bond to dentin, highlighting the importance of the right cementing agent in restorative dentistry.

## 1. Introduction

The digitalisation of technology for indirect dental restorations has led to the development of restorative materials [1,2]. The aesthetic materials used for indirect restorations include lithium disilicate ceramics, lithium disilicate zirconia-reinforced ceramics, zirconia ceramics, polymethyl methacrylate (PMMA) composites, and hybrid materials, which are composite materials with ceramic particles. They have different hardness, compressive strength, and varying adhesion to hard dental tissues (HDT), among other properties [2,3].

The new materials for digital technologies present dentists with questions related to the choice of material, particularly in terms of aesthetics and mechanical resistance, for indirect restorations [4,5,6]. Nowadays, with the development of novel 3D printing materials, these can be used for permanent restorations. From a clinical point of view, it is interesting to compare the strength of adhesive bonding of new 3D printing materials with milling materials. For the durability of aesthetic indirect restorations, the accuracy of the fabricated indirect restoration and its cementation are important. The cementation of indirect restorations is critical. The cement bonds two inherently different structures: the restorative material and the HDT (enamel and dentin). Cementation is influenced by two key factors: the treatment of the internal surface of the restoration and the type of cementing agent used. Different methods are used to treat the internal surface of the restoration: acid etching (phosphoric, hydrofluoric), sandblasting, use of silane, tribochemical treatment (coating the surface with a silica-based agent), laser treatment, and mechanical scraping [7,8,9]. The material for an indirect restoration determines the method for processing its internal surface.

For fixing aesthetic materials, the use of appropriate cement and adherence to a precise handling protocol are important [10]. Composite cement is preferred for cementing aesthetic indirect restorations [11,12,13]. Composite cement, according to the working protocol, is categorised as total-etch (etch-and-rinse type), self-adhesive, or self-etching cement [14].

Total-etch composite resin cement has the highest bond strength. Excellent retention and superior mechanical properties have been found. However, composite cements used with total-etch protocols on hard dental tissues have been found to cause postoperative sensitivity. Their handling protocol is highly sensitive to moisture and desiccation. The outcome of their application depends on operator skill, restoration design, and intraoral conditions [15].

Self-adhesive composite resin cements are a relatively new generation of composite cementing agents. They have a simplified handling protocol and are widely used. They were developed as a result of clinicians’ desire for simplified handling in cementing constructs [15]. They exhibit good adhesion to enamel and dentin, as well as to porcelain and composite restorations, without the need for additional adhesive agents [12]. In their initial phase of polymerisation, they have the properties of self-etching materials, i.e., they have low pH and high hydrophilicity, which leads to surface demineralisation [12]. Self-adhesive composite resin cement incorporates new methacrylate monomers with phosphoric acid groups, like 10-Methacryloyloxydecyl dihydrogen phosphate (10-MDP), which react with hydroxyapatite in the teeth to enable a self-adhesive reaction between this type of cement and the tooth structure. The polymerisation reaction is of the free radical type and is initiated by a photoinitiator or redox system [16]. According to the information provided by the manufacturers, these cements contain acidic and hydrophilic monomers that both demineralise and infiltrate the enamel and dentin, creating a strong bond. Multiple lines of evidence have demonstrated that, at a specific concentration, acid monomers effectively demineralise dentin and enamel, thereby facilitating adhesion to dental tissues through a micromechanical process [17,18]. However, the concentration of the acid monomers must be high enough to ensure adequate demineralisation and bonding to enamel and dentin, and low enough to avoid additional hydrophilicity in the polymerised material [19]. Unlike other types of composite cement, self-adhesive composite resin cement does not require treatment of the tooth surface, thus reducing technique sensitivity and manipulation time. Regarding the strength of the bond between self-adhesive composite cement and dental materials for indirect restorations, the literature is conflicting.

Self-etching composite resin cements were designed to meet the increased demand for cementation of metal-free restorations. In addition to the composite cement itself, adhesive agents are applied to the enamel and dentin. They have improved mechanical properties for the cementation of metal-free restorations, yielding good long-term clinical results [20]. Self-etching composite resin cement uses an acidic primer to process the tissues. They offer a rapid handling protocol, making them easier to use and reducing the risk of errors [21]. Difficulties in isolating the operative field and subgingival cavities have been cited as a disadvantage [22]. Clinical studies have shown that they have a weaker bond to the enamel compared to those using total etching. Self-etching composite cements are used to fix crowns and bridges where dentin is predominantly retained; inlays and onlays are fabricated for significant clinical crown defects and compromised retention [23]. Self-staining cements bond more strongly to dentin and less strongly to enamel [23]. They cannot completely strip the collagen fibres, which is why special functional monomers (10-MDP) are needed to improve adhesion [23]. 10-MDP is contained in the primer and can increase the adhesive bond strength.

The adhesive bond strength is important for the durability of indirect restorations. From a clinical point of view, it is interesting to compare the adhesion of self-adhesive and self-etching resin cement, containing 10-MDP. This research aims to investigate whether multi-step systems and the application of a primer affect adhesion. In addition, the aim of this study is to compare the adhesive bond of newly introduced materials for milling and 3D printing. The objective of this study was to evaluate the bonding strength of self-adhesive and self-etching resin cements to dentin for indirect restorations. The null hypothesis was that there would be no difference in the shear bond strength of self-adhesive and self-etching resin cements for indirect aesthetic restorations to dentin.

## 2. Materials and Methods

**Figure 1:**
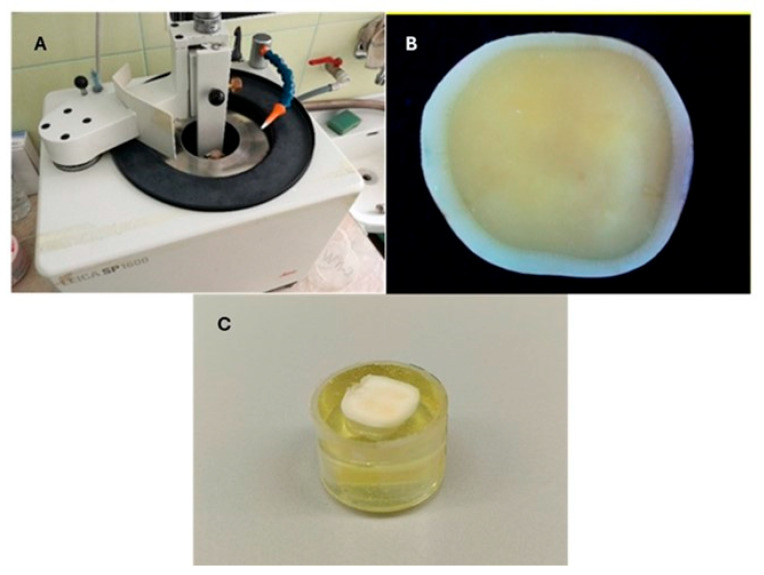
(**A**) General view of the Leica SP 1600 microtome (**B**). Prepared dentin plate for cementation. (**C**). View of the packaged dentin plate.

The Medical University–Sofia Research Ethics Committee approved this project (approval number: 5). For the purpose of the study, the following materials for indirect restorations were used (Table 1):

Twenty cubes of each material were made into tiles measuring 4 mm × 4 mm, with a thickness of 4 mm. They were fixed with two types of composite cement (Table 2) onto 80 extracted dentin molar plates (Figure 1).

### 2.1. Dentin Specimen Preparation

Eighty intact, healthy third molars extracted for orthodontic reasons were selected for the study. The teeth were cleaned with an ultrasonic device to remove soft tissues and examined under 9× magnification using a Leica S6 stereomicroscope (Leica Microsystems, Wetzlar, Germany). Teeth with defects such as hypoplasia and fractures were removed. The selected samples were stored in a 0.1% thymol solution. Radiographs were taken of the prepared teeth. The obtained data were used to calculate the distance from the occlusal surface to the plane through which the section should pass. Cutting was performed using a Leica SP 1600 microtome (Leica, Wetzlar, Germany) with the calculated distances (Figure 1A). The process was carried out in three stages—making cuts along the conditional planes ZX, ZY, and XY at 600 rpm and under continuous water cooling.

Dentin specimens were obtained (Figure 1B) and stored in water at 37 °C in a thermostat. The prepared samples were immersed in cylindrical holders measuring 5 mm × 4 mm, with their root part inward to about two-thirds of the crown. They were cast with modified epoxy resin KANOPKOS MS21 (Specialty Polymers Ltd., Sofia, Bulgaria; based on KER 828, Kumho P&B Chemicals, Seoul, Republic of Korea). Samples of dentin with a flat surface were obtained, referred to as dentin specimens.

### 2.2. Preparation of Samples from Different Materials

**Figure 2:**
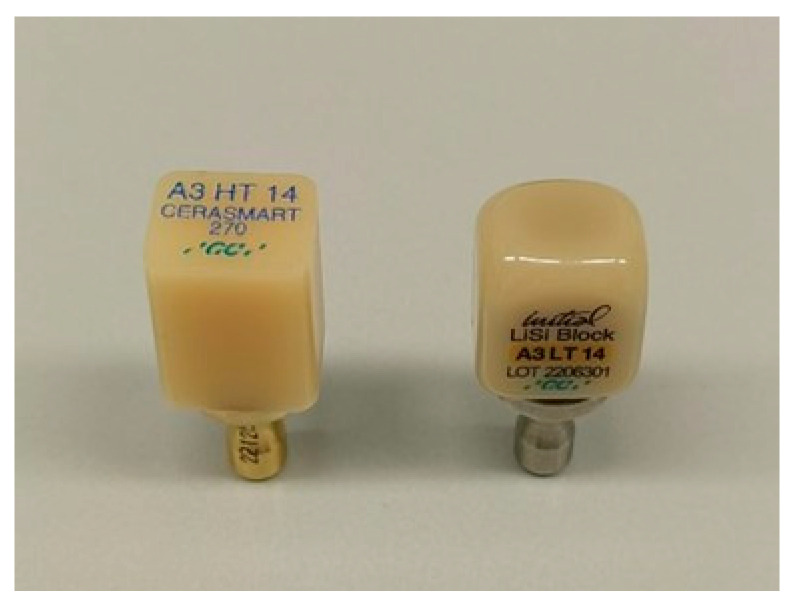
Blocks of Cerasmart 270 (**left**) and Initial LiSi (**right**).

The first two materials (Cerasmart 270 and Initial LiSi Block) were in rectangular shape (Figure 2).

The available metal bases, onto which the materials were glued, were used for reliable fixation in the cutting machine. After positioning in the microtome in the conditional planes ZX, ZY, and XY and under continuous water cooling, cuts were made to form cubic bodies with approximate dimensions of 4 × 4 × 4 mm. Test bodies of 20 pieces each were made from Cerasmart 270 (GC Europe) and Initial LiSi Block (GC Europe). Digital cube-shaped samples with dimensions of 4 × 4 × 4 were created using the 3D modelling software Meshmixer, software version 3.5.0. The obtained image was sent to a milling machine, and 20 reference models were made from the Katana ZR Noritake—a disk with a diameter of 98 and a height of 18 mm. The last series of test bodies was made using 3D printing technology from Crowntec NextDent, again with the same dimensions as the others. The cubic test bodies were stored in an insulated, humid environment at room temperature. The prepared samples were cemented to the dentin surfaces and distributed into eight groups of 10 samples per group, depending on the material and cement:

From group 1 to group 4, the cementing composite material was self-adhesive—i-CEM (KULZER, Germany).

Group 1: Cerasmart 270/i-CEM/dentin;

Group 2: Initial LiSi Block/i-CEM/dentin;

Group 3: Katana Zr Noritake/i-CEM/dentin;

Group 4: Crowntec NextDent/i-CEM/dentin.

From group 1a to group 4a, the cementing composite material was self-etching—Panavia V5 (Kuraray Noritake).

Group 1 a: Cerasmart 270/Panavia v5/dentin;

Group 2 a: Initial LiSi Block/Panavia v5/dentin;

Group 3 a: Katana Zr Noritake/Panavia v5/dentin;

Group 4 a: Crowntec NextDent/Panavia v5/dentin.

Depending on the investigated materials and cementing agents, the surfaces were treated according to the manufacturer’s recommendations and the data found in the literature:

- Dentin—cleaned with H_2_O_2_ and alcohol.
- Samples of Cerasmart 270 and Crowntec NextDent—sandblasting with Al_2_O_3_ and silane, Silan IT (ITENA, Paris, France).
- Samples of Katana Zr Noritake—sandblasting with Al_2_O_3_.
- Samples of Initial LiSi Block—hydrofluoric acid (HF) and silane.

**Figure 3:**
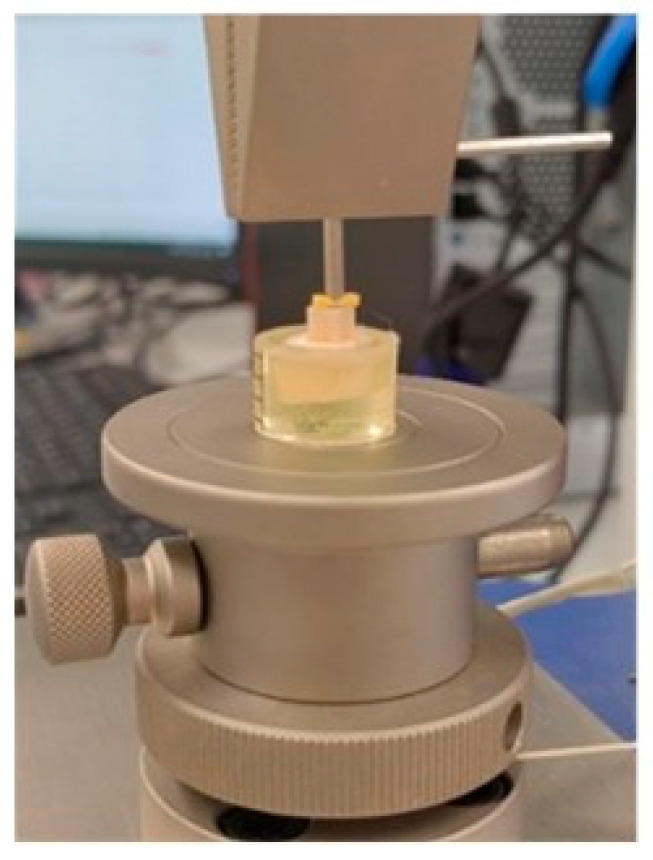
Cementation of materials to the dentin.

During cementation, a uniform, standardised pressure of 5 N was applied to the samples, and polymerisation was initiated from each side for 40 s. In the technological implementation of adhesion, the above-mentioned inevitable deficiencies are overcome by applying a constant force that presses the two adherents together. In this study, a new approach was proposed and applied. The control to ensure reproducible force was carried out using a software-controlled electromechanical stand for physical–mechanical testing (Multitest 2.5-i, Mecmesin, Slinfold, West Sussex, UK) (Figure 3). The possibility of implementing a specially programmed mode of operation was utilised. For reproducibility at this stage of the protocol for creating the adhesive bond, the start of photo-initiation was set to 2 s after reaching the selected constant force on the forming structure of the cube and dentin. For the samples that were fixed using self-etching cement, a primer—Clearfil for the ceramic surface and Tooth Primer for the dentin—was initially used, after which the cementing agent was applied, and the same pressure was again applied.

### 2.3. Method for Physical–Mechanical Testing of Shear Bond

**Figure 4:**
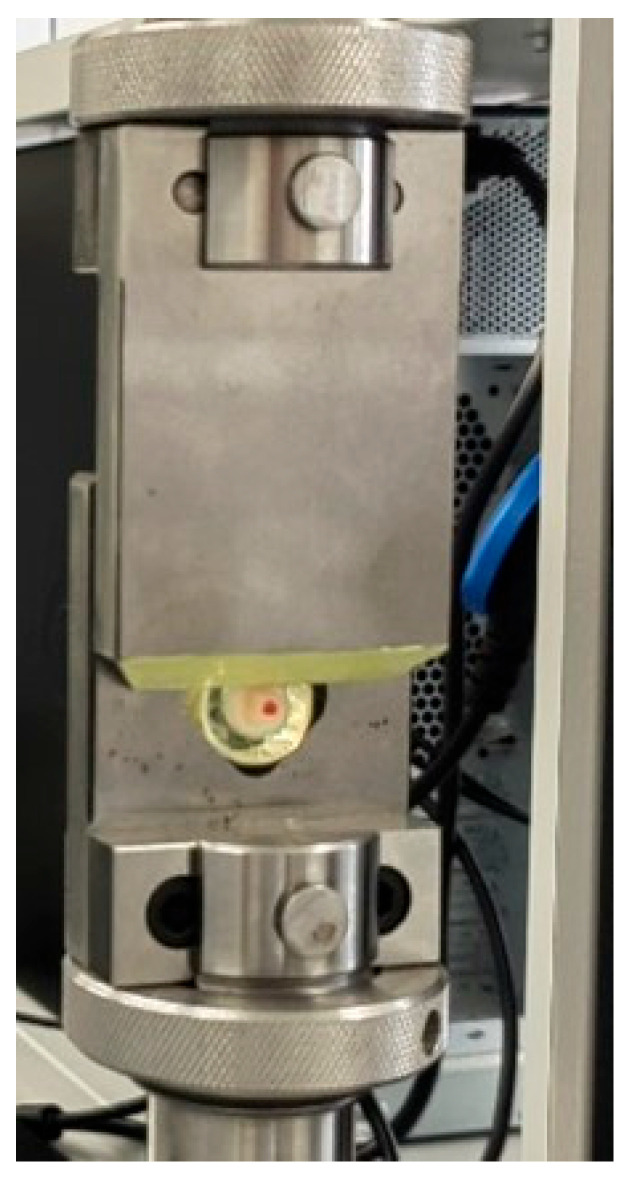
Microshear bond strength test of the samples.

The stand for physical–mechanical testing was electromechanical and equipped with software control. As a construction, it consisted of a single column with an integrated screw-driven drive, which drives a specially extended forward arm. A strain gauge cell was attached to it, which measured the magnitude of the force applied at one end. The positioning precision was 0.01 mm, and the force measurement precision was 0.1 N, with a range of up to 1000 N. The speed of arm movement could be selected in the range of 1 to 1000 mm/min. Depending on the direction of movement, tensile and compressive forces could be applied. The device for testing strength under shear loading consisted of two metal plates that slide over a common plane (Figure 4). One of them, marked as “frame,” had a hole cut in the shape of a pentagon. This standardised the size of the secured test body without limiting its shape. The other plate had a bevel on its short side and was therefore marked as a “knife.” The two parts of the device were secured in a standard way, but independently of each other, to the available connections of the stand for physical–mechanical testing. The speed and force of the pressure exerted by the knife were programmed in advance. All tests were conducted at a knife movement speed of 1 mm/min. The rate of recording the registered force was 10 Hz, and its accuracy was 0.1 N.

Initially, a static contact pressure of 0.5 N was applied. A control check by the operator regarding the functionality of all systems for conducting the test followed. Confirming this circumstance (in the dialogue mode of the software) started the testing process. In the interval from this initial moment to the mechanical destruction of the construction, the software automatically recorded pairs of data from the registered resistance force and the displacement of the knife. The maximum recorded force value was accepted as the breaking force of the test body.

The maximum shear stress characterising the strength of the specific adhesive bond was calculated using the formula:

τ = F/S

where

τ is the maximum tangential stress of the adhesive bond, measured in [MPa];

F—the maximum measured force up to the moment of destruction [N];

S—the area of the adhesive contact [mm^2^].

After shear strength testing, three types of fractures were observed:

- Adhesive-type—between the cement and dentin/material surface (≥70% of the total area of the two surfaces is not covered by cement);
- Cohesive-type—in the cement (≥60% of the total area of the two surfaces is covered with cement);
- Mixed-type—those that do not fall into the first two groups.

### 2.4. Scanning Electron Microscopy Evaluation

To determine the type of fracture at the contact boundary between the material and dentin, a stereomicroscopic study was conducted using a Leica S6 stereomicroscope (Leica Microsystems, Wetzlar, Germany) and scanning electron microscopy (SEM) with a Hitachi TM-400 tabletop device (Hitachi, Tokyo, Japan). The samples for the SEM study were coated with colloidal gold, and the study was conducted under conditions of 15 kV voltage and 1000× magnification.

### 2.5. Statistical Analysis

The mean values of µSBS data in MPa and their corresponding standard deviations were determined, and a Q-Q diagram was used to determine whether the data distribution was normal. A parametric test for checking the difference between more than two independent samples (ANOVA) was used when the distribution of shear strength in the samples was normal. Levene’s test for equality of variances was used to check whether the dispersion among the materials was equal. When the dispersion among the materials was equal, the Bonferroni test was used. When the dispersion among the materials was not equal, the Games–Howell test was applied. This test also determined the type of Student’s *t*-test used, a parametric test for assessing the difference between two independent samples (Student’s *t*-test). All statistical methods used were tested at a significance level of 99%. Statistical methods were applied using IBM SPSS Statistics 26, and graphical representations were created with Excel 2010.

## 3. Results

### 3.1. Quantitative Evaluation Results

**Figure 5:**
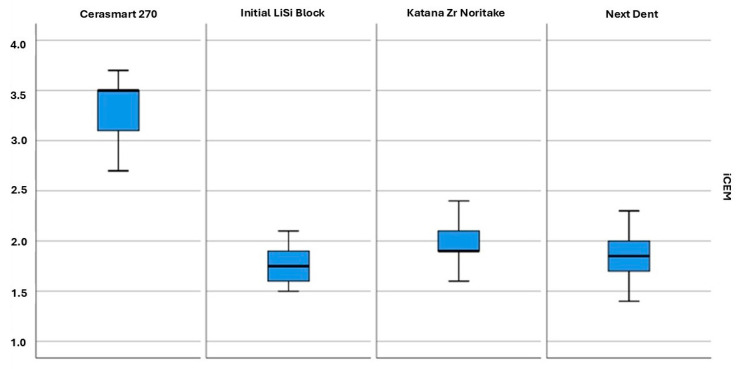
Shear bond strength box plot of i- CEM group. There was a statistically significant difference between Cerasmart 270 and Initial LiSi Block (*p* < 0.01), Cerasmart 270 and Katana Zr Noritake (*p* < 0.01), and Cerasmart 270 and Next Dent (*p* < 0.01). There was not a statistically significant difference between Initial LiSi Block, Katana ZR Noritake, and Next Dent (*p* > 0.01).

**Figure 6:**
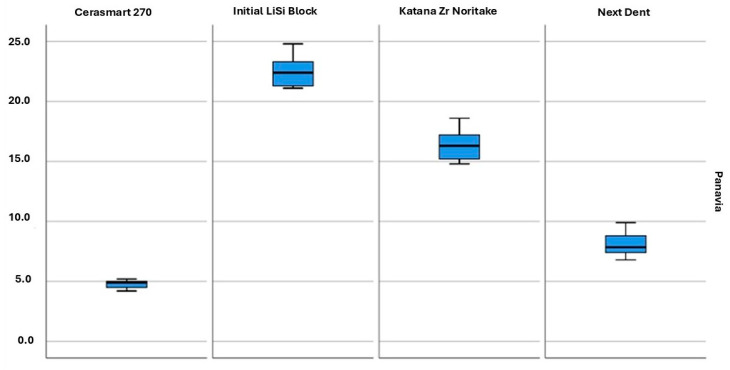
Shear bond strength box plot of the Panavia V5 group. There was a statistically significant difference between all tested groups (*p* < 0.01).

The mean values and standard deviation of shear strength in all groups are presented in Table 3 and Figure 5 and Figure 6. The values in the four groups, cemented with self-adhesive and self-etching cements, as well as for each material cemented with both types of cements, were compared. The highest values were observed in the group of LiSi Initial samples with Panavia V5 (22.6 ± 1.4), while the lowest values were recorded for LiSi Initial with i-Cem (1.8 ± 0.2).

When comparing the shear strength values between the groups cemented with the same cement, it was found that shear strength for all materials was normally distributed; therefore, a parametric analysis of variance (ANOVA) was performed to compare differences between more than two independent samples. Since a difference was found, a post hoc test was applied to determine in which groups these differences were observed. For materials with i-CEM cement, the dispersion in the groups, as determined by Levene’s test, was equal; therefore, a post hoc test was performed using the Bonferroni test. For materials with Panavia V5 cement, the dispersion in the groups based on Levene’s test was different, and therefore, the post hoc test was performed with the Games–Howell test. The test results are presented in Table 3.

When comparing the shear strength values between groups of the same material cemented with different types of cement, it was found that shear strength for all materials was normally distributed. Therefore, a parametric Student’s *t*-test was used to compare the two independent samples. Levene’s test showed that only the dispersion for material Cerasmart 270 between the two types of cement was equal. For the remaining materials, the dispersion between the two cements was different. The test results are presented in Table 3. When comparing the different materials cemented with i-CEM, it was found that the shear strength values for Cerasmart 270 were statistically significantly higher than those for the other materials in this group. No differences were found among the other materials.

The significance level between the materials with Panavia V5 cement was also less than the risk of error α = 0.01. Therefore, the alternative hypothesis was accepted that there were differences in the mean shear strengths between the individual materials. The post hoc test showed that this difference was significant among all materials, indicating that each material had a significantly different average shear strength compared to the others. The highest value was for the material Initial LiSi Block, and the lowest was for Cerasmart 270.

When examining the shear strength of groups of the same type of material cemented with both cements, it was found that the shear strength of each of the tested materials was higher with Panavia V5.

### 3.2. Qualitative Evaluation Results

Results for the type of failure from the stereomicroscopic studies are shown below.

**Figure 7:**
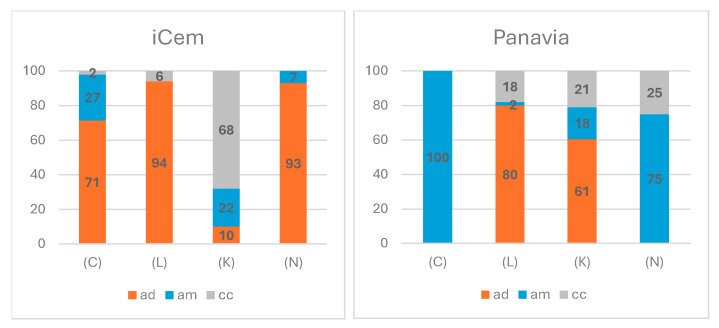
Failure mode percentages. C—Cerasmart 270; L—Initial LiSi Block; K—Katana ZR; N—Crowntec NextDent; ad—adhesive failure cement/dentin; am—adhesive failure cement/material; cc—cohesive failure in luting cement.

Figure 5 illustrates the data on the type of fracture after stereomicroscopic examination. To confirm the results, SEM was also performed (Figure 7). In all examined groups, an adhesive-type fracture was observed. Regardless of the cementing agent, no cohesive type of fracture was observed for the material Cerasmart. For samples from Initial LiSi and Katana, mixed fractures were observed, comprising both adhesive and cohesive types. The cohesive type of fracture predominates in the samples of Katana cemented with iCem. An interesting fact is that in the groups of samples of Cerasmart and NextDent cemented with iCem, the adhesive type of fracture was towards the dentin, whereas with Panavia cement, it was towards the material. There was a difference in the type of fracture depending on the material used.

**Figure 8:**
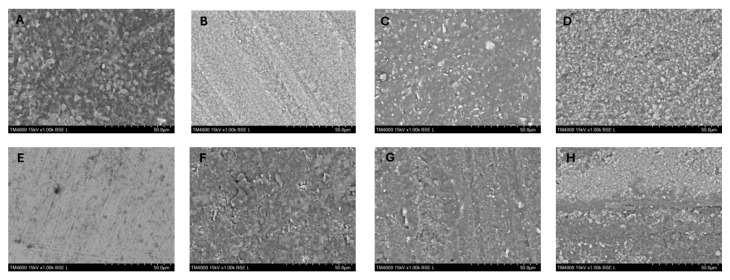
Results from SEM. (**A**) Adhesion fracture to dentin in group Cerasmart/i-CEM. (**B**) Adhesive fracture to dentin in group Initial LiSi Blok/i-CEM. (**C**) Adhesive fracture to dentin in group Katana ZR Noritake/i-CEM. (**D**) Adhesive fracture to dentin in group Crowntec Nextdent—i-CEM. (**E**) Adhesive fracture to material in group Cerasmart 270/Panavia V5. (**F**) Cohesive fracture in cement in group Initial LiSi Blok/Panavia V5. (**G**) Cohesive fracture in cement in group Katana ZR Noritake/Panavia V5. (**H**) Mixed fracture in group Crowntec Nextdent /Panavia V5.

The SEM analysis was performed at magnifications of 500× and 1000×. The samples cemented with self-adhesive cement most often showed adhesive-type fractures towards the dentin. In the groups where self-etching cement was used, mainly cohesive-type fractures were observed in the cement, which also explains the higher shear strength values (Figure 8).

## 4. Discussion

The cementing agent connects two different structures—hard dental tissues (dentin and enamel) and restorative materials. The restorative material must be pre-treated and prepared in a specific way to achieve reliable adhesion [24]. Various methods for treating the restorative material have been described and studied in the literature—etching with hydrofluoric acid, sandblasting, silane coating, and laser treatment [8,24,25]. In this study, two types of composite cement were examined—self-adhesive and self-etching. They have been recently developed, and no adverse consequences after cementation, such as postoperative sensitivity, have been reported [23,26].

To evaluate the strength of the adhesive bond, the shear strength test is most commonly used [20]. In this type of study, it is not necessary to cut the cemented surfaces to obtain samples, as is done in the micro tensile strength test. This reduces the risk of pre-stress occurrence [27,28]. In this study, we examined the shear strength between zirconia ceramics, lithium disilicate, PMMA, and composite material with ceramic particles and dentin, cemented with composite self-etching and self-adhesive cement.

The self-etching composite cement Panavia V5 contains special functional monomers such as 2-hydroxyethyl methacrylate (HEMA) and/or 10-Methacryloyloxydecyl dihydrogen phosphate (10-MDP), which enhance the strength of the adhesive bond, improve the diffusion and penetration of other monomers, and provide antimicrobial action [27]. 10-MDP can bond with enamel and dentin, forming MDP-Ca salts chemically. In this way, both micromechanical and chemical bonding are achieved [23,26,27]. MDP-Ca salts exhibit high stability, resistance to hydrolysis, and long-lasting durability, thereby providing stability to the adhesive bond in an aqueous environment. 10-MDP is part of the primer for conditioning hard dental tissues, but it is not a component of the cement itself [27]. Pimental and co-authors indicate that 10-MDP creates a chemical bond between the materials and dentin [27].

In this study, the materials cemented to dentin using self-etching material showed statistically significantly better shear strength. Pimental, Inokoshi, De Angelis, and Naliboglu obtained similar results [27,29,30,31]. The protocol for the research on Panavia V5 includes the application of two primers: Clearfil Primer and Tooth Primer. Both contain 10-MDP.

Cerasmart is a hybrid material that contains ceramic particles and a polymer matrix. In the conducted study, a statistically significant difference in shear strength was found between the groups using self-etching and self-adhesive cement, favouring self-etching. Similar results were reported by Cekic-Nagas et al. [32]. They obtained values close to ours, noting that self-etching cements using a primer with 10-MDP have statistically significantly better shear strength. Cekic-Nagas et al. [32] reported the presence of adhesive-type fractures similar to those found in this study. Since they did not cement blocks to the dentin surface, the fractures were only between the material and the cement.

Lithium disilicate is a silica/glass ceramic [33,34]. The silane groups in the composite cement composition chemically bond with the glass particles in the material [35]. Lithium disilicate was treated with hydrofluoric acid before cementation to create a micromechanical bond [35]. In the groups where self-adhesive cement was used, bond failure was again observed between dentin and cement. In the group of samples fixed with self-etching cement, we found cohesive-type fractures in the cement. The shear strength towards dentin was statistically significantly stronger for lithium disilicate when using the self-etching composite cement Panavia V5 compared to the self-adhesive cement i-CEM. Similar results were obtained by Naliboglu and co-authors [31].

When comparing the results obtained for the zirconia ceramic Katana Zr Noritake, a statistical difference was reported between the groups cemented with i-CEM and Panavia V5. The shear strength obtained with the self-etching cement Panavia V5 was statistically significantly better. We also observed cohesive-type fractures, which is favourable with regard to the strength of the bond between dentin and zirconia ceramic. The reason is the functional monomer (containing 10-MDP) from the ceramic primer, which interacts chemically with the zirconia surface, improving wetting and chemical bonding to zirconia [27]. This results in an increase in the shear strength between the two materials. The self-adhesive cement i-CEM does not contain MDP. Moreover, the dentin surface was not treated before cementation. This is a prerequisite for the occurrence of a weaker bond between the cement and dentin. Literature data from other conducted studies confirm our results [30]. Serichetaphongse et al. also reported higher shear strength values when using Clearfil primer, as well as cohesive-type fractures [36]. De Angelis et al. reported results similar to ours and stated that the best adhesion was achieved by using the self-etching cement Panavia V5 and Clearfil primer [29]. They observed that mixed-type fractures were most often observed in the group of samples with Panavia V5, unlike our study. The reason could be due to a difference in the study’s methodology. The authors cemented the zirconia tiles to zirconia samples rather than to dentin.

The printing material Crowntec NextDent is relatively new, and we did not find studies in the literature regarding the shear strength between it and dentin samples. In this study, higher values were recorded when cementing with self-etching cement, similar to all other groups—the samples from Crowntec NextDent bonded better than those from Cerasmart 270. In the group of Crowntec NextDent fixed with self-etching cement, we found a predominance of mixed-type fractures, which are also considered acceptable [37]. The relatively good shear strength results give us reason to use the material for permanent restorations.

In the groups of samples cemented with i-CEM, a statistically significant difference was found between the samples from Cerasmart 270 and all others. The hybrid ceramic bonds statistically better with i-CEM. In the groups of samples cemented with Panavia V5, a statistically significantly higher shear strength was found compared to the self-adhesive cement for all tested materials. The best result was shown by the lithium disilicate, followed by zirconia, composite, and the weakest result—hybrid ceramic. Elsaka et al. also reported weaker bonding of hybrid ceramics compared to glass ceramics [38]. Naiboglu et al. [31] confirmed these results. Flury et al.’s results contrast with these findings and did not show a statistically significant difference [39]. According to Naiboglu, the difference between the groups was attributed to microstructural characteristics, including the type and percentage content of filler, material structure, and mechanical properties [31].

The type of cement used significantly influences the results in our study. Multi-step systems, in which a separate primer is applied, increase the strength of the adhesive bond. Despite the more difficult work protocol, they are recommended in clinical practice.

In our study, the type of fracture of all samples was determined using a stereomicroscope, as described in most of the literature [27]. Some of the samples were also examined using scanning electron microscopy to verify the results. The type of fracture was used to measure the success of the adhesive bond strength [40]. Adhesive fractures are defined as unacceptable, mixed as acceptable, and cohesive as ideal [41]. Additional comparisons between the two composite cements reveal specific features. In the case of using iCem, adhesive failure at the dentin interface was consistently observed, whereas with Panavia, a share of failure was always attributed to the material. Regarding cohesive failure, it was absent only for Nextdent Crowntec when using iCem, whereas with Panavia, this type was not observed. Further comparisons revealed that only the combination of Cerasmart and Panavia V5 had 100% adhesive-type failure towards the material.

Comparisons between the shares of different types of failures within the group of composite cement iCem reveal the characteristics of the interactions between the different elements of the bonded restorations. For example, in the combinations Cerasmart, LiSi, and Nextdent Crowntec, adhesive failure was observed by both mechanisms. The high share of adhesive fractures towards dentin, combined with low strength indicators, suggests the need for an additional protocol for treating the dentin surface. As noted above, the application of Panavia to the same materials has a different effect. The reduced adhesion to Cerasmart is a basis for recommending specific pre-treatment of the bonding surface in this specific case. Similar reasoning and recommendations can also be applied to the case of Crowntec; however, in this instance, there is a compensating effect from a larger share of cohesive-type fractures. The increased area of cohesive-type failure, as well as the relatively small shares of adhesive-type failure towards the material in LiSi and Katana, provides grounds to assume that these distributions play a decisive role in the significant increase in the final bond strength. For self-etching cement, significantly higher shear strength values were recorded. Similar to us, other authors have also found that at lower values, adhesive fractures prevail, while at higher values, cohesive or mixed fractures occur [27,37,42,43].

Despite well-documented results, several limitations of this study should be mentioned. Further research should include different resin-based and glass-ionomer cements. Future studies should use conditions that mimic the oral environment, such as cyclic loading. In addition, the bond strength of different materials to enamel could be investigated.

## 5. Conclusions

Within the limitations of this laboratory investigation, it can be concluded that the cement type significantly influences bond strength, and the null hypothesis was rejected. The self-etching Panavia V5 demonstrated significantly higher results than self-adhesive resin cement i-CEM. The fractures that occur at the dentin–cement–material interface with self-adhesive cement were predominantly adhesive. The fractures that occur at the dentin–cement–material interface with self-etching cements were cohesive or mixed type.

## Figures and Tables

**Table 1:** Materials used in the study.

Materials (Manufacturer)	Composition	Procedure/Surface Treatment
Cerasmart 270 (*GC Europe*, *Leuven*, *Belgium*)—milling CAD/CAM block	71% Silica and barium glass nanoparticles, Bis-MEPP, UDMA, DMA	Sandblasting 50 µm Al_2_O_3_ + silane
Initial Li Si Block (*GC Europe*, *Leuven*, *Belgium*)—milling CAD/CAM block	55–80% SiO_2_, 10–30% Li_2_O; 5–20% other oxides; pigments: trace	5% HF + silane
Katana Zr Noritake (*Kuraray Noritake*, *Ins*, *Tokyo*, *Japan*)—milling CAD/CAM disk	3 mol % Y2O3 tetragonal zirconia polycrystals	Sandblasting 50 µm Al_2_O_3_
Crowntec NextDent (*SAREMCO Dental AG*, *Rebstein*, *Switzerland*)*—3D printed material*	30–50% silanised dental glass, pyrogenic silica, 4,4′-isopropylidiphenol, ethoxylated and 2-methylprop-2enoic acid, initiators.	Sandblasting 50 µm Al_2_O_3_ + silane

Abbreviations: Bis-MEPP—bisphenol A ethoxylate dimethacrylate; UDMA—urethane dimethacrylate; DMA—dimethylacetamide.

**Table 2:** Composition of resin-based cements used.

Cement	Manufacturer	Composition	
*i-Cem* *self-adhesive resin cement*	KULZER, Hanau, Germany	Paste	51 wt% Di-, tri-, and multifunctional acrylates, initiators, stabilizers, 49 wt% filler
*Panavia V5* *self-etching* *resin cement*	Kuraray Noritake, Tokyo, Japan	Clearfil primer	3-MPS, ethanol, 10-MDP
		Tooth Primer	10-Methacryloyloxydecyl dihydrogen phosphate (MDP), 2-Hydroxyethyl methacrylate (HEMA), hydrophilic aliphatic dimethacrylate, accelerators, water.
		Paste	Bis-GMA (bisphenol A-glycidyl methacrylate), TEGDMA (Triethylene glycol dimethacrylate), hydrophobic aromatic dimethacrylate, hydrophilic aliphaticdimethacrylate, initiators, accelerators, silanated barium glass filler, silanated fluoroaluminosilicate glass filler, colloidal silica, silanated aluminum oxide filler, dl-camphorquinone, pigments.

**Table 3:** Mean ± standard deviation of shear bond strength (SBS) values in MPa for each material with different cements.

Luting Agent	CERASMART 270	Initial LiSi Block	Katana Zr	NextDent
i-CEM	3.3 ± 0.3 ^A,a,^*	1.8 ± 0.2 ^A,b^	2.0 ± 0.2 ^A,b^	1.9 ± 0.3 ^A,b^
PANAVIA V5	4.8 ± 0.2 ^B,a^	22.6 ± 1.4 ^B,b^	16.4 ± 1.3 ^B,c^	8.1 ± 0.9 ^B,d^
*p*-value (in column)	*p* < 0.01	*p* < 0.01	*p* < 0.01	*p* < 0.01

* Different superscript uppercase letters in each column for each material indicate significant differences (*p* < 0.01). Different superscript lowercase letters in each row for each cement indicate significant differences (*p* < 0.01).

## Data Availability

The original contributions presented in the study are included in the article, further inquiries can be directed to the corresponding author.
